# Towards reducing chemical usage for weed control in agriculture using UAS imagery analysis and computer vision techniques

**DOI:** 10.1038/s41598-023-33042-0

**Published:** 2023-04-21

**Authors:** Ranjan Sapkota, John Stenger, Michael Ostlie, Paulo Flores

**Affiliations:** 1 Present Address: Center for Precision and Automated Agricultural Systems, Washington State University, 24106 N. Bunn Rd, Prosser, WA 99350 USA; 2 Agricultural and Biosystems Engineering, North Dakota State University, 1221 Albrecht Blvd, Fargo, ND 58102 USA; 3 NDSU Carrington Research Extension Center, Carrington, ND 58421-0219 USA

**Keywords:** Environmental sciences, Engineering

## Abstract

Currently, applying uniform distribution of chemical herbicide through a sprayer without considering the spatial distribution information of crops and weeds is the most common method of controlling weeds in commercial agricultural production system. This kind of weed management practice lead to excessive amounts of chemical herbicides being applied in a given field. The objective of this study was to perform site-specific weed control (SSWC) in a corn field by: (1) using a unmanned aerial system (UAS) to map the spatial distribution information of weeds in the field; (2) creating a prescription map based on the weed distribution map, and (3) spraying the field using the prescription map and a commercial size sprayer. In this study, we assumed that plants growing outside the corn rows are weeds and they need to be controlled. The first step in implementing such an approach is identifying the corn rows. For that, we are proposing a Crop Row Identification algorithm, a computer vision algorithm that identifies corn rows on UAS imagery. After being identified, the corn rows were then removed from the imagery and remaining vegetation fraction was classified as weeds. Based on that information, a grid-based weed prescription map was created and the weed control application was implemented through a commercial-size sprayer. The decision of spraying herbicides on a particular grid was based on the presence of weeds in that grid cell. All the grids that contained at least one weed were sprayed, while the grids free of weeds were not. Using our SSWC approach, we were able to save 26.2% of the acreage from being sprayed with herbicide compared to the current method. This study presents a full workflow from UAS image collection to field weed control implementation using a commercial size sprayer, and it shows that some level of savings can potentially be obtained even in a situation with high weed infestation, which might provide an opportunity to reduce chemical usage in corn production systems.

## Introduction

Corn (*Zea mays* L.) is a major cereal crop that accounts for more than 95% of total feed grain production in the United States (US) and provides food, feed, and fuel for people and livestock worldwide^1^. The importance of corn lies not only in its high yield potential but also in its diverse uses, including as a source of starch, sweeteners, biofuels, and industrial products^2,3^. Corn is a staple food for many people, especially in developing countries, where it provides a significant portion of the daily calorie intake. In addition, corn is a vital component of animal feed, providing essential nutrients for livestock such as cattle, pigs, and poultry^4^. Moreover, corn has a significant role in the biofuel industry, where it is converted to ethanol and other biofuels^5^. This renewable energy source contributes to reducing greenhouse gas emissions and decreasing dependence on fossil fuels^6^. Corn cultivation also has numerous environmental benefits, such as reducing soil erosion and improving soil quality^7^. Given the importance of corn in food, feed, fuel, and industrial production, the sustainable and efficient management of corn cultivation is crucial for ensuring global food security, reducing greenhouse gas emissions, and promoting sustainable agriculture.

The current corn production system uses excessive amount of chemical herbicide for weed control. About 1 billion pounds of conventional pesticides are used each year to control undesirable vegetation such as weeds in the agricultural production system of the United States (U.S.), which amount to a cost of almost $9 billion^8,9^. One of the major reasons for excessive use of chemicals in agriculture is the conventional method of spraying herbicides for weed control, often called a blanket application, which accounts for 59% of the major pesticide expenditures in economic crops in North America^10^. The blanket application implies spraying herbicide uniformly across the field without considering the spatial distribution of the weeds, which often causes overuse of chemical herbicides.

Chemical herbicides are widely used in modern agriculture to control weeds, protect crop yields, and improve farming efficiency. Herbicides reduce competition for resources such as water, nutrients, and sunlight, promoting crop growth and leading to higher yields^11^. Additionally, they can prevent the spread of diseases and pests that may harm crops, thus improving food quality and safety^12^. However, overreliance on herbicides can have negative environmental consequences, such as contamination of soil and water resources and the development of herbicide-resistant weeds. In addition to being harmful to the environment^13,14^, chemical herbicides can be harmful to human health as they have been linked to cancer^15^, attention deficit hyperactivity disorder (ADHD)^16^, alzheimer’s^17^, DNA damage, cardiovascular diseases, neurological disorders, and reproductive disorders^18^. Moreover, excessive chemical use in agriculture has the potential to harm nervous system, endocrine system, and reproductive system of human^19^. Furthermore, excessive use of chemicals in agriculture have become one of the major reason for surface and groundwater pollution^20–23^, becoming a threat to the ecosystem and public health. Although organic farming is seen by many as a potential solution for the excessive use of chemicals in agriculture, it is not a silver bullet to meet the United Nations sustainable development goals by 2030^24^. Therefore, to maintain environmental sustainability and human health, agricultural production systems are in dire need of technologies that can significantly reduce the amount of chemicals currently being used, without compromising crop yields.

An alternative way to neglect chemical use for weed control in agricultural production system is through physically removing weeds by using robotics platforms. Some of the latest automated robots such as the autonomous weed robot by “ecoRobotix” (ecoRobotix, Vaud, Switzerland) and “Deepfield Robotics” (Deepfield Robotics, Renningen, Germany), use ground-based machine vision and image processing techniques to perform site-specific weed control (SSWC) in row crops^25^. However, these robotic solutions are not effective to be deployed in a large commercial agricultural field (hundreds or thousands of acres). To overcome that issue, some companies have developed and commercialized solutions such as “Weedseeker$\circledR$” (Trimble agriculture, California, USA), and “WEED-it” (WEED-IT, Steenderen, Netherlands), which use optoelectronic sensors to measure the reflection intensity of vegetation, allowing them to discriminate vegetation from soil background^26^. The shortfall of those products, at the time of this writing, is that they cannot discern weeds from crops early in growing to allow such technologies to be used for weed control. Recently, John Deere (Illinois, USA) launched See & Spray Ultimate$\circledR$, a sprayer that enables detection and control of weeds during the growing season in corn, soybean, and cotton. More details regarding the field performance of that technology remain to be seen since the sprayers fitted with the system will be available only in 2023^27^.

Another approach of performing SSWC is possible through the use of remote sensing technology to accurately map the weed distribution information across a field and integrate that with a spraying platform^28^. The main idea here is to spray herbicide only to those areas where weeds are present^29^. To accomplish that, one would need a high-resolution imagery to discern plants growing in the field as weeds and crop. Since satellite imagery would present some challenges to discern weeds from crops, due to its limitations in providing adequate spatial resolution^30^, the use of UAS with high-resolution cameras seems to be a promising platform, which can be processed and analyzed to obtain an accurate weed distribution map from a field^31^. For that approach to work, is imperative to detect weeds accurately early in the growing season. In most crop weed management programs, weed treatment is recommended at the early growth stages of both crops and weeds. Mapping weeds at that stage can be a challenging task because of four main reasons: (1) weeds are not uniformly distributed across the field, which necessitates working at a single pixel size on the image^32^, (2) crop and weeds have the same or similar reflectance properties, (3) interference of soil background^33^, and (4) very small size weeds which might not captured by camera^34^.

One of the earliest studies related to SSWC using UAS^35^ reported about the possibility of the use of UAS imagery and imagery analysis techniques to perform accurate discrimination between weeds and sunflower at early growth stages, which is key to implement any SSWC. More recently, the use of UAS in agriculture for SSWC have become a topic of several publications in specialized literature related to agriculture^36–44^. Around the same time, more sophisticated and powerful image analysis algorithms, such as machine learning (ML) and deep learning (DL) have being developed and adapted for agricultural applications, and they have gained space as effective ways to process large amounts of data in agriculture^45,46^.

Another study conducted by Peña et al.^37^ developed an object-based image analysis (OBIA) procedure to generate a weed map in an experimental maize field in Spain using a six-band multispectral camera. The OBIA procedure classified crop rows, discriminated crops and weeds based on their relative positions to crop rows, and generated a weed infestation map. The study achieved an accuracy of 86% in generating weed map, however, the study was limited to virtual simulation, and further field implementation is necessary to validate its effectiveness. Likewise, Hunter et al.^47^ investigated the efficiency and efficacy of an unmanned aerial vehicle integrated system (UAV-IS) for weed management compared to ground-based broadcast applications. The UAV-IS was found to be more efficient at identifying and treating target weedy areas while minimizing treatment on non-weedy areas. However, it treated 20–60% less area than the ground-based applications and missed up to 26% of the target weedy area. The study concludes that UAV-IS has the potential to improve integrated pest management programs and reduce herbicide resistance, but battery replacement issues need to be addressed for commercial farming use. Similarly, a recent study by Jensen et al.^48^ presented an approach for automated site-specific fallow weed management using unmanned aerial vehicles (UAVs) and precision spray technology. The system employs machine vision and GIS technology to detect and map green weeds, which are subsequently targeted for precision herbicide application. Although field evaluation of the system showed some herbicide savings, the accuracy varied for different weed species, and some species were even not identified with good accuracy. This suggests that the system may not be suitable for managing all weed species in fallow fields. Future work is needed to optimize the system’s efficiency and commercial viability.

Identification of crop rows in an UAS imagery using ML and DL have become another subject for several recent studies^49–58^. Although, most of these studies have been able to identify crop rows, and discriminate between weeds and crops during the early stage, they were limited to virtual simulations only. Most of these reports regarding the use of UAS imagery for SSWC fall short of providing a real word solution for SSWC, since most of them just report on the capability of different algorithms to separate weeds from crops. Moreover, most of these studies regarding crop row detection, were able to identify the rows as lines with great accuracy^51–58^, however, the authors could not provide any insights on how to use those detected crop rows in practical agriculture. Additionally, most of the reports are performed in a small area of agricultural land which would probably raise questions about the research validity in bigger agricultural area.

Corn is a major crop for the state of North Dakota (ND), US. In 2020, ND farmers grew corn in 1.5 million hectares of corn in ND^59^. Weed competition is a major cause of corn yield losses in ND, and chemical herbicide is the most widely used option for weed control^60^. In order to optimize weed control efficacy, the use of pre-and post-emergence chemical herbicide treatments has been proven to be the most effective weed control strategy^61,62^. At present, most of the farmers apply a blanket application of herbicides for post-emergence weed control in corn, with no consideration regarding the spatial distribution of weeds across the field. That application often results in an overuse of chemical herbicides. The cost of post-emergence herbicide application (product + application) in ND is usually around $8-12/ha (2020 growing season). If one could use UAS imagery to generate a weed distribution map, implement SSWC, and apply chemicals to only those areas where weeds are present in the field, that could lead to significant savings on chemicals for corn growers. Using the area grown with corn in 2020 in ND and the cost associated with the chemical application, those savings could reach as much as $5.5–7.5 million, with as little as a 10% reduction in applied acreage. In addition to the financial savings, there are environmental savings, which are harder to put a number to it, that can be realized using applying fewer chemicals to the land.

This research is embodying the following contributions, as no previous study has reported on the whole workflow, from image collection to weed control application in the field, to implement SSWC in corn by integrating UAS imagery with a commercial-size sprayer:

- Development and validation of a Crop Row Identification (CRI) algorithm for UAS-based imagery to accurately identify corn rows.
- Mapping of post-emergent weed infestation in the corn field and creation of a grid-based weed prescription map for site-specific weed control.
- Integration and field implementation of the prescription map through a commercial-size sprayer.
- To compare the amount of chemical herbicide applied using our proposed approach (SSWC) with a traditional herbicide application method.

## Methods

**Figure 1:**
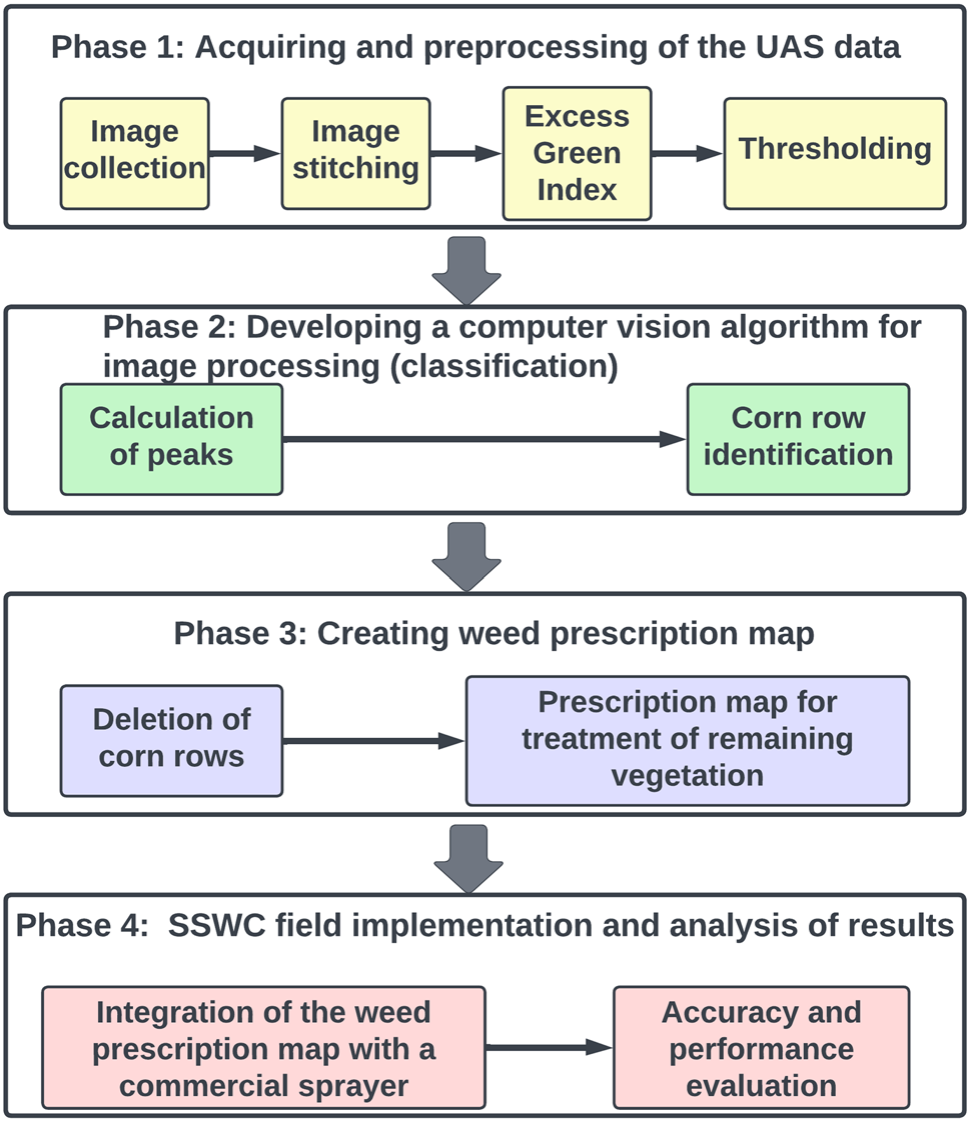
Workflow diagram followed to implement a site-specific weed control approach in a corn field.

This study can be divided into four phases (Fig. 1); (1) acquiring and preprocessing UAS data, (2) developing a computer vision algorithm for corn row classification, (3) creating a weed prescription map and (4) field implementation of the prescription map through a commercial-size sprayer.

### Study site

The study was carried out in a corn field of around 17 hectares (ha), located at Carrington, North Dakota $\left( 47.51^{\circ }\,\textrm{N}, 99.12^{\circ }\, \textrm{W}\right)$. The experimental area for this study was provided by North Dakota State University (NDSU) Carrington Research Extension Center (REC). According to the Web Soil Survey (WSS), the soil in the experimental field was composed of Heimdall and similar soil (42%), Emrick and similar soil (37%), and minor components 21%. The field was planted with silage corn on May 12, 2021, with a 30-inch row spacing. Prior to planting, on May 7, the field received a pre-emergence herbicide treatment (Verdict^63^, (0.98 kg/ha), which was applied at a rate 140.3 L/ha.

### Data collection

**Figure 2:**
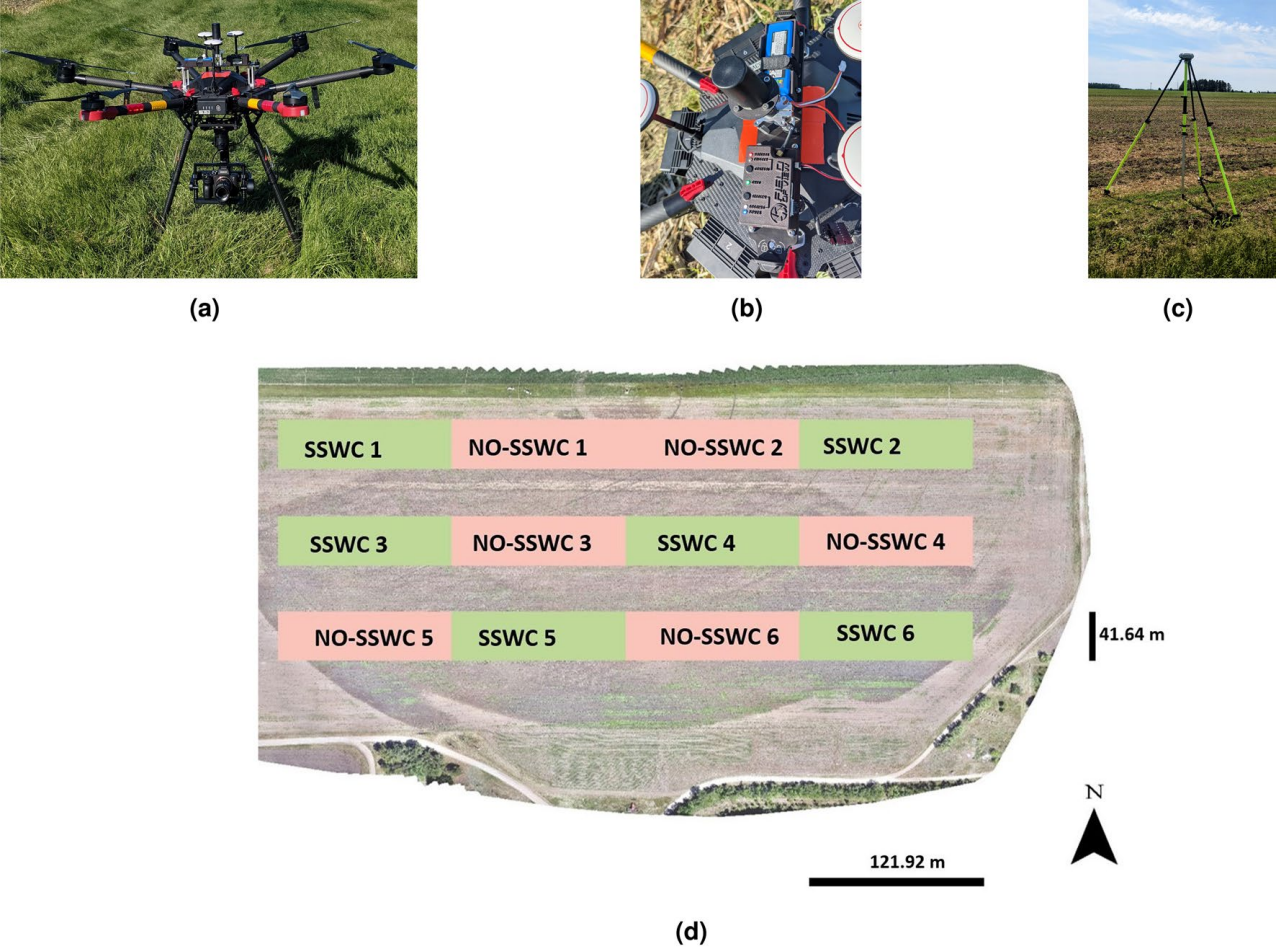
(**a**) DJI Matrice 600 Pro with a Sony Alpha 7R II, 42.4-megapixel RGB camera and Geosnap PPK system (top) mounted to it; (**b**) detailed view of Geosnap PPK system mounted on top of the aircraft; (**c**) iG4 GNSS base station that was kept in ground for stationary position; and (**d**) Corn field orthomosaic showing the placement of the SSWC (green) and no-SSWC (pink salmon) treatment plots in the experimental area.

The UAS flights for image collection were carried out on June 14, 2021. A DJI Matrice 600 Pro (M600) (Fig. 2a)(DJI, Shenzhen, China), outfitted with a Sony Alpha 7R II 42 Megapixel RGB camera (Sony City, Tokyo, Japan) was used to capture the aerial images of the field (Fig. 2d). The camera has a 7952 $\times$ 5304 pixels (42.4 megapixels) sensor resolution, and a focal length of 35mm. Integration unit for the camera on the drone was made by FieldofView LLC (Fargo, North Dakota, USA), which manufactures a device called GeoSnap PPK (Fig. 2b) that allows one to trigger the camera and geotag the images with PPK (post-processed kinematic) accuracy (2 cm). Since distance between the research field and nearest CORS (Continuously Operating Reference Stations; Cooperstown, ND) was too far, which would cause a degradation of the geotag accuracy, an iG4 GNSS (Global navigation satellite system) base station (iGage Mapping Corporation, Salt Lake City, USA) (Fig. 2c) was used to implement the PPK correction to the images’ geotags. The UAS was flown autonomously using flight missions created in Pix4DCapture app (IOS version) (PIX4D, Prilly, Switzerland). Flights were carried out at 107 meters approximately above ground level (AGL), with the camera at nadir position, with 75% overlap both front and side, and the UAS speed was adjusted (by the app) to allow 1.4 seconds interval between pictures. Altogether, three flights were carried to cover the experimental field, collecting a total of 2251 images.

All the data processing in this study was performed in a desktop computer that have Windows 10 Pro, version 20H2, a 64-bit operating system with 132 gigabytes of RAM. The desktop was provided with two switchable GPUs. One was Intel(R) UHD Graphics 630, and the other was NVIDIA GeForce RTX 2080 SUPER.

### Image preprocessing and stitching

**Figure 3:**
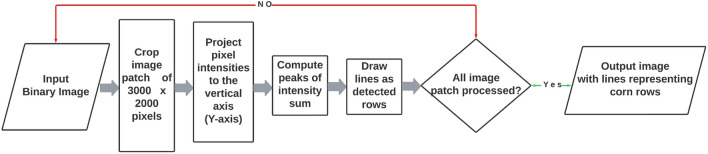
Flow diagram of Corn Row Identification (CRI) algorithm to detect lines over corn rows in binary imagery generated from UAS imagery.

In order to generate accurate (2 cm accuracy for latitude and longitude, and twice that for elevation) geotag information for each image, the information collected by the GNSS base station and GeoSnap device were processed using EzSurv software (Effigis, Montreal, Canada), which provided an output as “.csv” file with post-corrected geotag information for each picture. That file was then used during the images stitching process. Images were processed into an orthomosaic (Fig. 2d) using Pix4DMapper (Pix4D, Prilly, Switzerland). It took 6 h and 25 min for the software to generate the output as orthomosaic, which had an average ground sample distance (GSD) of 0.63 cm/pixel. That orthomosaic served as basis for all the subsequent analyses to implement our SSWC approach. Figure 3 shows the orthomosaic with the experimental units where both the SSWC and conventional (NO-SSWC) treatments were applied.

### Vegetation identification

In order to segment the vegetation fraction from the background on the orthomosaic, excess green index (ExG) was calculated using Eq. (1).

1 $$\begin{aligned} ExGI = 2g-r-b ; where\, (r=\frac{R}{R + G + B}\, g =\frac{G}{R + G + B}\, b =\frac{B}{R + G + B} ) \end{aligned}$$

where r, g, and b are the normalized values of the bands red, green, and blue respectively, and R, G, and B represent the pixel digital number values of the red, green, and blue color bands respectively.

Once the ExGI was calculated, a threshold value (0.08) was applied to distinguish all green vegetation from the background. Since the objective of threshold selection is to choose a threshold value that can effectively distinguish the object of interest (in this case, green vegetation or corn rows) from the background, the threshold value of 0.08 was selected after manually trying different values in ArcGIS Pro software (ESRI, Redlands, United States) to find the optimal threshold that covers the maximum portion of green vegetation in the field during the early corn growing season.

The optimal threshold value may vary depending on several factors such as image quality, lighting conditions, and the characteristics of the object being segmented. Therefore, it is common practice to manually or automatically test different threshold values and evaluate their performance based on a specific criterion, such as the percentage of pixels classified correctly or the accuracy of the segmentation result. Once the threshold value was applied to the ExGI values to segment the image into the binary foreground (vegetation) and background (non-vegetation) region, it created a binary imagery with pixel intensity value 1 for the vegetation and 0 for the remaining background as described in Eq. (2):

2 $$\begin{aligned} g(x,y) = {\left\{ \begin{array}{ll} 1, &{} \text {if } ExGI(x,y) > 0.08 \\ 0, &{} \text {if } ExGI(x,y) \le 0.08 \end{array}\right. } \end{aligned}$$

where ExGI(x,y) represents the pixel value of the Excess green index calculated from UAS imagery at position (x,y), and g(x,y) is the binary output image that results from applying the thresholding operation.

In this expression, if the ExGI value of a pixel (x,y) is greater than the threshold value of 0.08, then the corresponding output pixel g(x,y) is set to 1, indicating that it belongs to the green vegetation (corn rows). Conversely, if the ExGI value of a pixel (x,y) is less than or equal to the threshold value of 0.08, then the corresponding output pixel g(x,y) is set to 0, indicating that it belongs to the background (non-vegetation). This results in a binary image with pixel intensity value 1 for the vegetation (corn rows) and 0 for the remaining background. That binary image was further processed to identify the corn rows.

There are a few scientific reasons why the excess green index could be more useful than NDVI in this scenario. First, ExGI is less affected by soil reflectance than NDVI, which can be particularly important during the early growing season when crops are small and soil background is more prevalent in the imagery^64^. NDVI is known to be sensitive to variations in soil background, which can lead to false positives or negatives in vegetation segmentation. ExGI, on the other hand, is less sensitive to soil reflectance and can more accurately differentiate vegetation from the soil. Second, ExG can better differentiate between different types of vegetation than NDVI. This is because NDVI is sensitive only to the chlorophyll content in plants, which can be similar between different types of plants. ExGI, on the other hand, is more sensitive to differences in leaf structure and pigments, which can vary more between different plant species. In the case of small corn plants and weed vegetation, which may have similar chlorophyll content but different leaf structures, ExGI can more accurately separate the two.

### Algorithm development for crop row detection

We are proposing the use of a crop row identification (CRI) algorithm for detection of corn rows in the binary orthomosaic imagery. In order to expedite the processing time, the CRI algorithm was applied, using Python version only to the six SSWC treatment plots, which was around 30,500 square meters in area. Figure 3 shows the flow diagram for the CRI algorithm that is being proposed for corn row identification.

**Figure 4:**
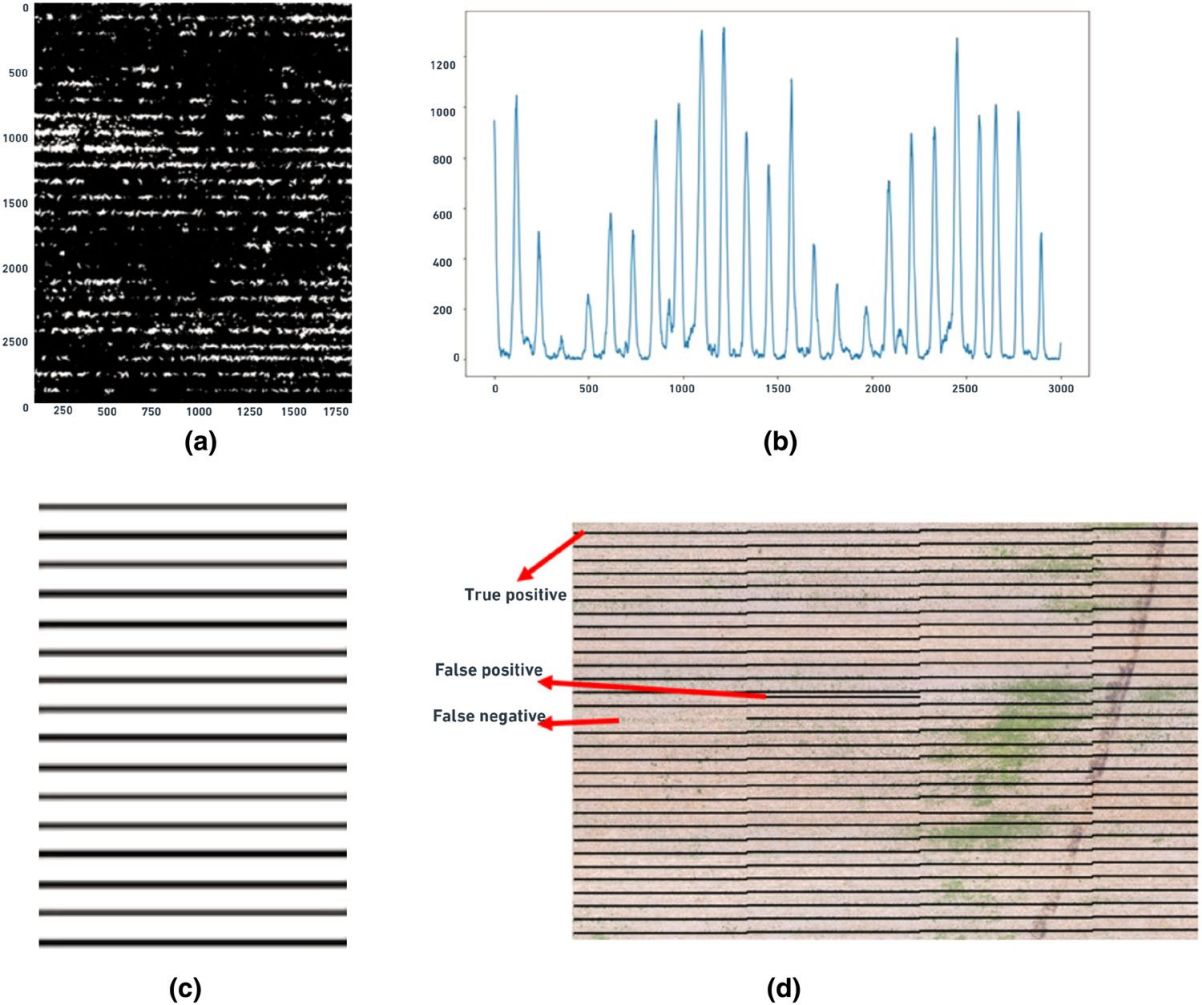
(**a**) A section of the binary ExGI imagery (3000 $\times$ 2000 pixels) showing the horizontal orientation of rows, (**b**) local maximums (or peaks) for each corn row on the section of imagery, (**c**) lines drawn over the each computed local maximum, and (**d**) Comparison of actual corn rows with the identified lines created by the CRI algorithm on a corn field imagery.

The first step in the procedure for line detection over the corn rows was to prepare a zero-like array using NumPy, which returned a new array of a given shape and type, filled with zeros. Once the zero-like array was created, a function was defined as a “cropped image” which cropped the input imagery into a smaller size (3000 $\times$ 2000 pixels). This function was set with a combination of NumPy-SciPy libraries. The NumPy variable “sum y” sums up all non-zero-pixel values into one value for each cornrow. The SciPy variable “peaks” calculated the local maximum values (peaks) in the array of Y-axis summed values. Pixel intensity along X-axis (columns), as shown in Fig. 4a, were summed and projected towards Y-axis (rows) in order to compute the local maximums (peaks) in Y-axis as shown in Fig. 4b. Once the local maximums were computed for each row, straight lines perpendicular to Y-axis were drawn as shown in Fig. 4c. The same process was repeated for all section that was inside the boundary of the SSWC treatment plots.

Once the lines were drawn over the computed local maximums in each row, those lines were visually compared with the original corn rows (ground truth) on the imagery using ArcGIS Pro to verify the performance of CRI algorithm for accurately identifying the corn rows from the UAS imagery. Figure 4d shows the visual comparison of the generated outputs as true positive (TP), false positive (FP) and false negative (FN). In this study, TPs are the total number of predicted lines that exactly laid over the actual corn rows. Likewise, there were some places where actual corn rows were present, but the algorithm did not generate line over that row. These kinds of cases were considered FNs. Similarly, there were some lines generated where there were not any corn rows, and the total number of those kinds of lines were considered as FPs. Also, since the background in the imagery was negative and the CRI algorithm did not detect any background as a background value, TN is zero.

### Weed mapping across SSWC treatment plots

**Figure 5:**
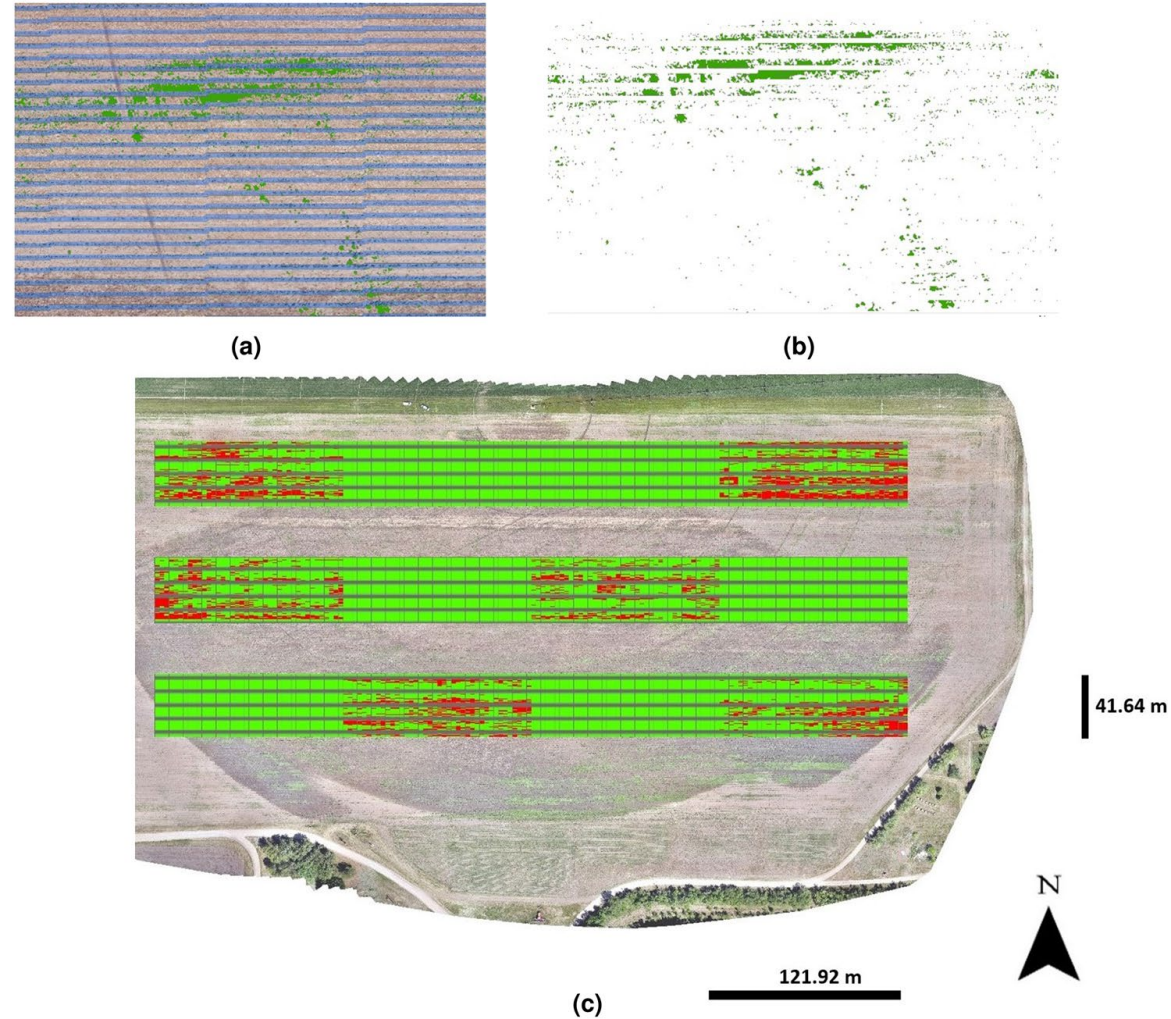
Buffered (3.5-inch on both sides) corn rows (**a**) that were removed from the imagery to create the weed map across the field (**b**) and the (**c**) variable rate prescription map (green cells = 140.3 L/ha and red cells= 0 L/ha) generated based on UAS imagery collected over a corn field using a Sony Alpha 7R II camera.

Our approach of mapping weeds is quite simple, and that is by design. All the vegetation growing within a certain distance (buffer) from the identified rows are considered corn plants, and the remaining of the vegetation, growing mainly between the rows, are considered weeds. Hence, once the corn rows were identified, the next step was to identify the vegetation fraction that would be classified as weeds. Our approach was to identify and delete all corn plants from the treatment plots, and the remaining vegetation was then classified as weeds. In order to implement that approach, we created a 3.5 inches buffer on both sides of the corn row lines as shown in Fig. 5a. This 3.5-inch buffer was wide enough to cover most of the corn plants, which were then deleted from the imagery, leaving behind the weeds present between the corn rows as shown in Fig. 5b. Since the weed pressure in the field was unusually high in the 2021 growing season, in order to implement the full workflow of the proposed approach for SSWC, from image collection to field spraying with a commercial size sprayer, the research team opted to overlay a grid of cells of 0.5 m $\times$ 3.04 m on the imagery. That resulted in an average value of 35% of the cells free of weeds across the SSWC treatment. The decision of spray or no spray on a given cell was based on the presence of weeds in a given grid cell. Grid cells that contained weed were assigned a rate of 140.3 L/ha herbicide mix solution, while the cells free of weeds were assigned at a rate of 0 L/ha, which is no-spray. The idea is that sprayer would just shut-off the nozzles while travelling over the no-spray cells. Figure 5c shows the prescription shapefile created in ArcGIS Pro, which was converted to a prescription map in a later step. The red cells among the replications of SSWC treatment were free of weeds, whereas the green cells contained weeds.

### Field area experimental design

To avoid an impact of irrigation on the results, the experiment was placed within the footprint of the center pivot irrigation system available for that field. A set of 24 plots, each measuring 121.9 m long by 41.6 m wide (the same width of the sprayer boom), were arranged in a 6 (North-South direction) by 4 (East-West direction) format placed over the experimental area. Next, every other row of plots were deleted (12 plots total), and the remaining plots were used for the experiment. For each to experimental units, treatments (SSWC and NO-SSWC) were randomly assigned, resulting in a complete randomized experimental design, with six replications across the field.

### Integration of weed map into a commercial sprayer

**Figure 6:**
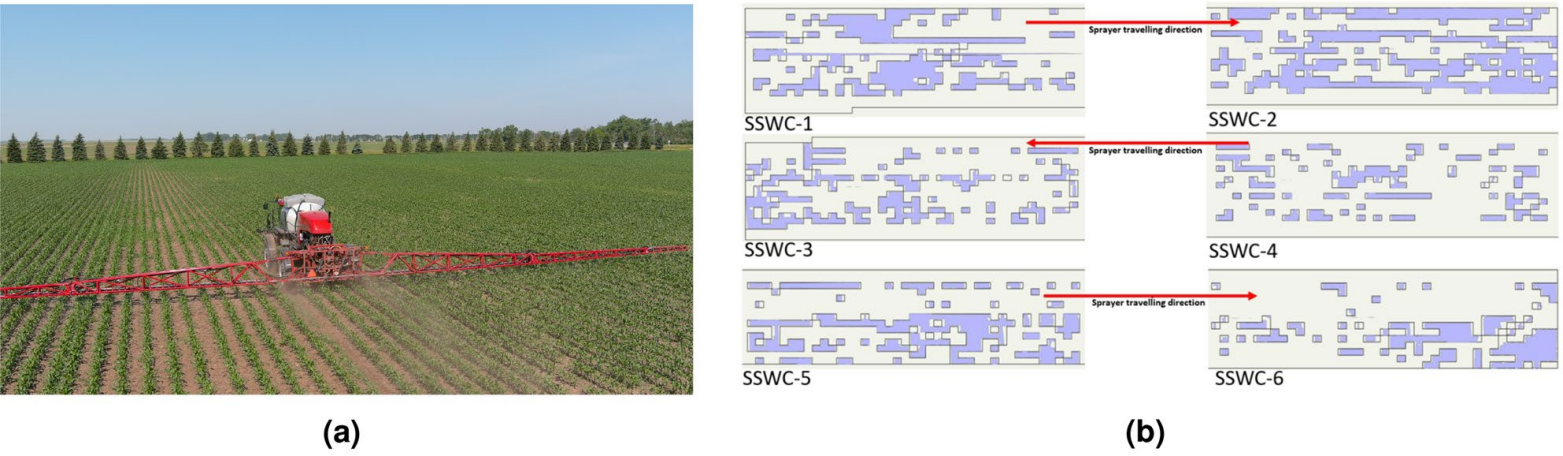
(**a**) Case IH Patriot 4440 series sprayer used to implement site-specific weed control in a corn field. The sprayer was equipped with an AIM command FLEX system, RTK GPS receiver, Viper 4+ cab computer, and 41.68 meter wide boom; (**b**) Overlap of Rx map, generated by AgSMS, where the regions enclosed by dark black lines where the no-spray regions (0 L/ha), and the as-applied map. Purple color regions are the area where the sprayer turned its nozzles off while operating at 10.5 km/hr speed (approximate), while all the remaining area was sprayed (140.3 L/ha).

A Case IH Patriot 4440 self-propelled sprayer (Racine, Wisconsin, USA), model year 2021, (Fig. 6a) was used to spray the experimental area. The sprayer was provided with an AIM command FLEX system, which was the latest technology from Case IH that enables consistent, flexible, and accurate spray application in commercial agriculture, regardless of speed and terrain. In addition, the system enhances control of liquid product flow and pressure more accurately than conventional rate controller and enables instant on/off of individual nozzles, with a nozzle valve diagnostic system.

The sprayer was set up with a Raven RX1 real time kinematic (RTK) receiver (Raven Technology, Sioux Falls USA), which operates at a frequency of 10 Hz. Following Case IH engineer’s advice, the application speed for this study was kept below 11 km/hr (10.5 km/hr actual speed application) to allow for the sprayer to maintain position accuracy resolution of at least 0.3 m (one US foot) per 1/10th of a second or 3 m (10 US feet) per second. That cab computer requires a certain folder structure, so it can read prescription maps (Rx) from an external storage device, such as a thumb drive. AgSMS Advanced software (Ag Leader, Iowa, USA) was used to read the shapefile created in ArcGIS Pro and to convert that file into a prescription map (Rx map) following the format required by the Viper 4+ computer cab. Once the Rx map was brought into the Viper 4+ display, the regions indicated by green color was set to be sprayed with a 140.3 L/ha, which is the same rate that farmers would use for a conventional chemical application.

### Assessing the spraying performance in terms of spray accuracy and chemical savings

Once the spraying operation was completed, an as-applied map was downloaded from the sprayer’s cab computer to compare with the prescription map in terms of spray and no-spray area. The spatial area recorded on the 6-SSWC treatment plots (Fig. 6b) of the as-applied map where the sprayer did not spray any chemical herbicide was calculated and compared with the other 6 plots (no-SSWC) where conventional method was applied.

Additionally, application accuracy of the sprayer was evaluated based on it’s ability to perform individual nozzle shut-off over the land where there was no presence of weeds. Based on the sprayed and not sprayed spatial area in the treatment zones between the prescription map and the as-applied map, the sprayer’s performance for chemical saving was evaluated. For that, the total area which was recorded as not-sprayed in the as-applied map was considered as Measured Value, and the total area which was set to be not-sprayed in the prescription map was considered as Expected Value. The accuracy of application in terms of spraying was calculated using Eq. (3):

3 $$\begin{aligned} Accuracy (\%) = \frac{Sum\ of\ Measured \ Value}{Sum\ of\ Expected\ Value \ n} \times 100\% \end{aligned}$$

Here, “n” represents the number of grid cells measured. The value of n is not explicitly present in this formula as it refers to the total number of grid cells measured, which is implicitly included in the Measured Value and Expected Value calculations for each treatment plots.

### Post-harvest field analysis

In order to investigate the experimental plots in terms of weed growth in a post harvest season, we collected a post harvest imagery on September 21, 2021, where image collection, image stitching, georeferencing, ExGI calculation, segmentation, and thresholding was carried out in a similar way as described for early-stage corn field data collection. The surface area of leftover and newly germinated weeds in the SSWC and conventionally approached test plots in the post harvest imagery was calculated and the datasets were analyzed using SAS PROC MIXED (SAS Institute, Cary, USA) with a mixed procedure using REML (restricted maximum likelihood) estimation. The area covered by weeds in the SSWC test plots was compared to the area of weeds present in six no-SSWC test plots using a pair-wise T-test with a significance level of 0.05 (*p* = 0.05).

## Results and discussion

### CRI Algorithm performance for crop row detection

The CRI (corn row identification) algorithm developed in this study demonstrated great accuracy in identifying corn rows from UAS imagery. The algorithm produced 2313 true positives, 12 false positives, 0 true negatives, and 8 false negatives values. The precision, recall, F1-score, and accuracy metrics were all above 0.99, indicating excellent performance in identifying corn rows from a 61,000 square meter land area. The algorithm processing time was 8 seconds on the previously described desktop computer. Additional details on classification results from the CRI algorithm are shown in Table 1.

**Table 1:** Classification results for the CRI algorithm in identifying corn rows.

	True positive	False positive	True negative	False negative
Corn rows	2313	12	0	8
Percentage	99.48%	0.05%	0.00%	0.34%
Classification metrics				
Precision	99.35%			
Recall	99.65%			
F1-score	99.49%			
Accuracy	99.01%			

Here, precision is the percentage of correctly identified corn rows out of all the rows identified as corn row, while recall is the percentage of correctly identified corn rows out of all the actual corn rows in the image. F1-score is the harmonic mean of precision and recall and provides a balanced measure of their performance. True positives (TP) are the correctly identified corn rows out of all the actual corn rows in the image, while false positives (FP) are the pixels identified as corn rows, but they are not actual corn rows. False negatives (FN), on the other hand, are actual corn rows that were not identified by the algorithm. The percentage of TPs and FPs provides insights into the accuracy of the algorithm in detecting corn rows. A higher percentage of TP indicates that the algorithm is correctly identifying corn rows, while a higher percentage of FP indicates the potential for over-detection, where non-corn pixels are falsely identified as corn rows. Over-detection can lead to unnecessary processing or misapplication of herbicides during weed management. In contrast, a higher percentage of FN indicates under-detection, where actual corn pixels are not identified by the algorithm. Under-detection can lead to missed opportunities for weed control or misapplication of herbicides leading to weed resistance.

**Figure 7:**
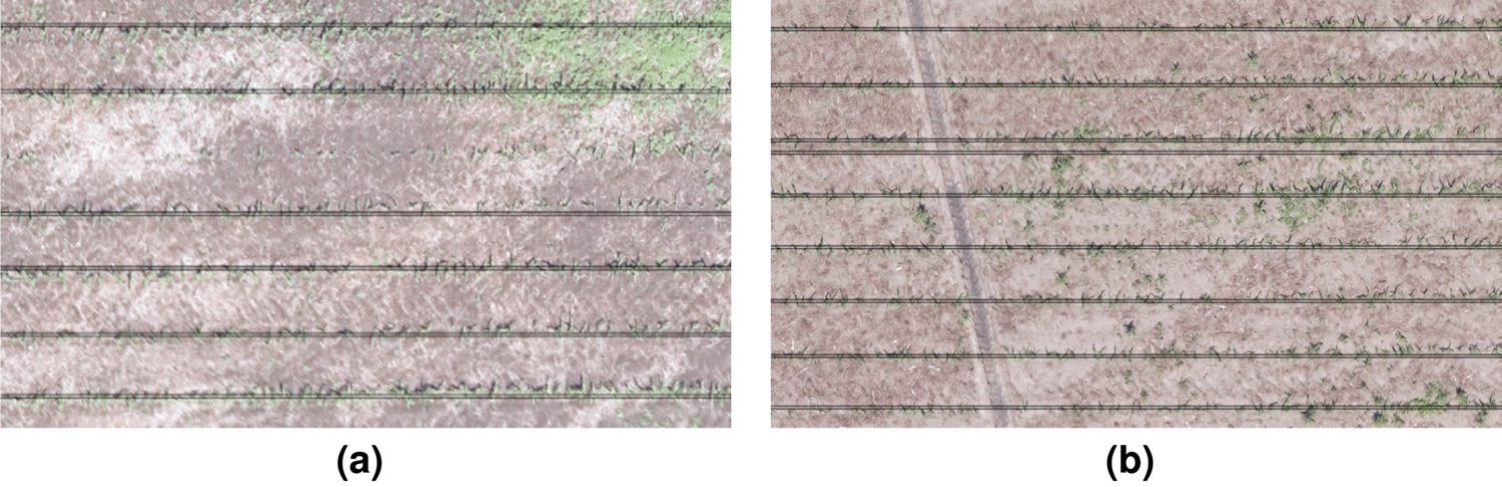
(**a**) Sample image where the proposed CRI algorithm failed to detect an existent corn row as a line on the ExGI imagery of a corn field, (**b**) Sample image where the proposed CRI algorithm detected two lines over a single corn row on the ExGI imagery of a corn field.

Because of the very small size of some corn plants as shown in Fig. 7a, the algorithm failed to identify line over those regions. The row of corn plants that could not be identified in the figure is due to the effect of the threshold value (0.08) that was applied during image segmentation. Use of higher resolution camera that can map even the smallest size of corn plant could possibly solve this issue. Also, in very few regions, we have noticed two lines representing a single corn row as shown in Fig. 7b. This is because the corn rows are not perfectly straight inside the region of 3000 $\times$ 2000 pixels, specified in the CRI algorithm. Since the corn row is not straight over the part of image, two small peaks(local maxima) have been identified in this region and hence, the algorithm displayed 2 lines to represent a single corn row. Setting up smaller region of interest during image processing could possibly solve this issue.

### Application accuracy and chemical savings using our SSWC method

**Figure 8:**
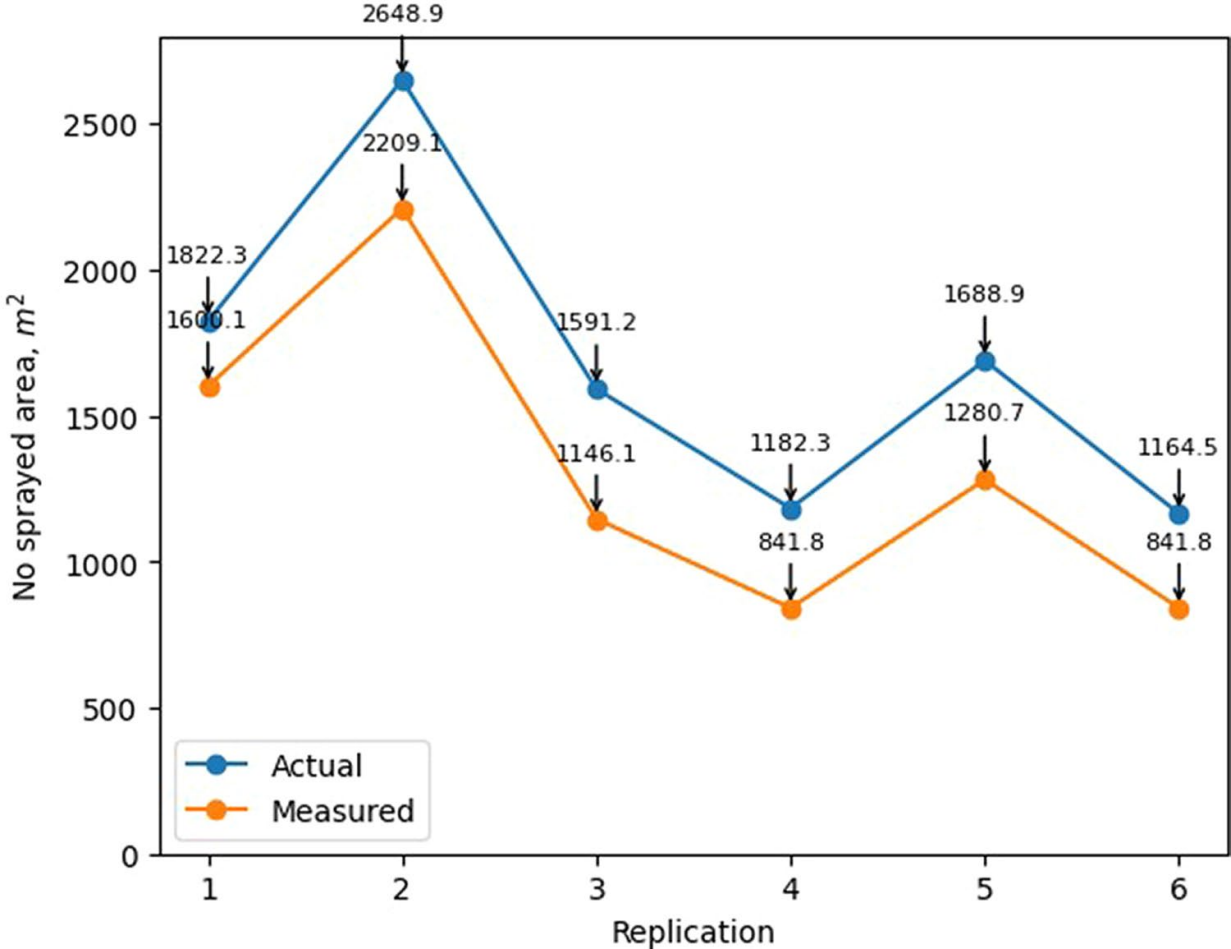
Comparison of the land area, in m^2^, between the prescription map uploaded to the sprayer (Actual) and area covered a during application (Measured) across the six replications for the site-specific weed control treatment.

Overall, the study was able to achieve an application accuracy of 78.4% in terms of not-spraying in the regions free of weeds. Figure 8 shows the no-spray area distribution information based on the prescription map (actual value) and on the as-applied map (measured value). As one take in consideration the results showing on Fig. 6b, the application accuracy seems to be largely affected by the start and stop of the spraying in each spraying area. One key factor that can be playing a role on those results is the speed of the sprayer, which was 10.5 km/h on this study. Additional studies should be conduct to evaluate the impact of the sprayer speed on the spraying accuracy.

**Figure 9:**
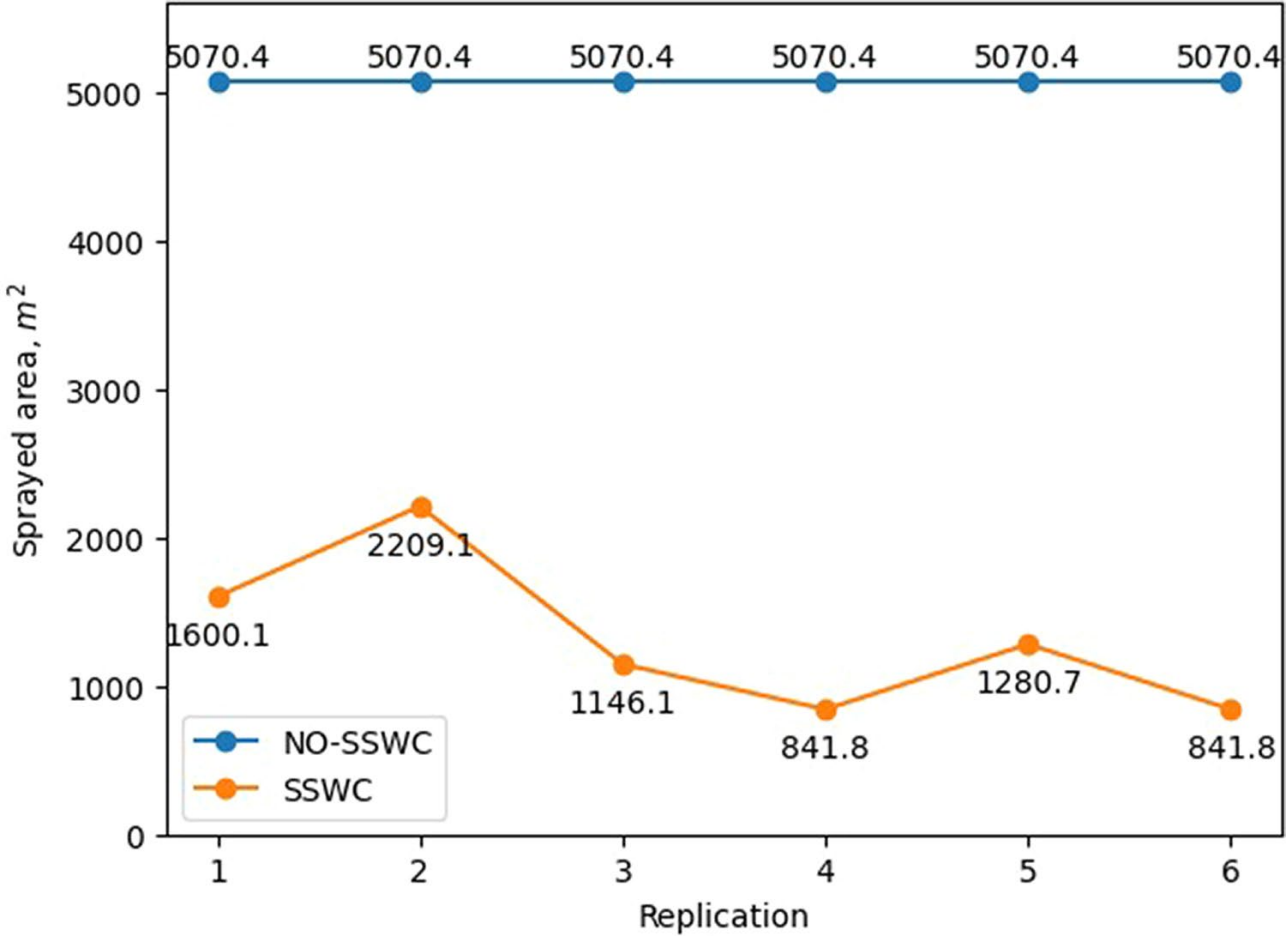
Comparison of the land area, in m^2^, not sprayed across replications for site-specific (SSWC) and non site-specific (NO-SSWC) weed control treatments.

Likewise, our approach for SSWC achieved significant chemical savings. Using the prescription map to turn off the sprayer’s nozzles on the grids cells free of weed resulted in savings of 26% of the area to be sprayed. Figure 9 shows the consumption of chemical herbicides, across all 6 replications, on the basis of land area using our SSWC approach and the conventional (no-SSWC) approach. Although the weed pressure at the season we performed this study was unusually high, the study proved that it is possible to spray chemicals only to the areas with weeds in the field. As an average, the SSWC treatment save around 26% of chemical herbicide in comparison with a blanket application (No-SSWC).

However, to achieve more efficient and effective herbicide application, reducing costs and environmental impacts while increasing crop yields, there are still opportunities for further improvement in the performance of the sprayer through the integration of remote sensing tools. One potential area for improvement is the integration of real-time sensors on the sprayer itself. This would enable the sprayer to adjust the rate of herbicide application in real-time based on the density of weeds detected by the remote sensing tools. For example, if a dense patch of weeds is detected, the sprayer could increase the rate of herbicide application in that specific area to ensure effective weed control. Another potential improvement is the use of more advanced remote sensing technologies, such as hyperspectral imaging or LIDAR. Hyperspectral imaging can provide more detailed spectral information about the crop and weeds, allowing for more accurate classification and mapping^65,66^. LIDAR can provide 3D imaging, allowing for more accurate detection of the height and density of vegetation, which can help in distinguishing between crops and weeds^67^. Furthermore, integrating machine learning algorithms into the remote sensing analysis can improve the accuracy of weed detection and classification. By training machine learning models on a large dataset of remote sensing data and ground-truthed weed maps, the algorithms can improve the accuracy of weed identification and provide more detailed information about the distribution of weeds in the field.

Likewise, in this study, we focused on mapping weeds between the corn rows using a simple buffer-based approach. However, we acknowledge that there may be some weeds present within the rows that are not captured by our method. To address this limitation, our original idea for the grid cell size is either 1.5 × 3.0 m (5 × 10 US feet) or 3.0 × 3.0 m (10 × 10 US feet). The first one would cover two rows of corn, while the bigger cell size would cover four rows. Since we make the spray or not spray decision based on the cell grid and not on the individual weeds, we believe the approach would control most of the weeds withing the rows of corn as well, since the approach would result in spraying two or four corn rows. Due to the high weed infestation in the experimental area, we use a smaller cell size to implement the whole workflow and to generate some saving in chemical. If we had used either a 1.5 × 3.0 m or a 3.0 × 3.0 m grid cell size for this study, approximately 100% would need to be sprayed, and not saving would be achieved.

Although some leading agricultural machinery manufacturing companies, such as John Deere and CNH Industrial, have announced about their future release of an automated sprayer which could perform site-specific application in some crops including corn, the adoption rate of the new technologies by farmers could be slow due to the expensive cost of agricultural spray machines. Our approach provide farmers that already a sprayer with individual nozzle control an option for site-specific weed control.

In a post harvest imagery based field evaluation of the SSWC and no-SSWC treatments plots, we found that the treatment were significantly different in terms of weed presence $\left( \textrm{F}_{1,5}=11.41,\; p<0.0197\right)$. The amount of weeds present in SSWC (3.4 m^2^) treatment plots in terms of surface area was 3.4 times higher than the amount of weeds that were present in no-SSWC plots (1 m^2^). The findings from this study could be a foundation to pursue various research questions that our study could not address, because of time and other limiting factors. Although the imagery obtained from the field had high resolution (GSD= 0.63 cm per pixel), we might have missed smaller weeds than the camera resolution during the data collection of the early growing season. Those weed detection skips might be one of the reasons why we observed weeds in the SSWC plots after the corn harvest. One could argue that image resolution should be increased to avoid those skips, but there are other challenges to achieve that, such as cost of better hardware (UASs and sensors), time to collect data (by flying lower with the same equipment used on this study), and time to process the data (lower altitude flight would lead to many more pictures captured, which increases processing time). The reasons to why the SSWC had more weeds than no-SSWC could likely be a combination of factors: (1) weeds were sprayed too late (rate not adequate to the size of the weeds); (2) lack of overlap when individual nozzles were applying chemical (applying even less chemical to the already too big/tall weeds); and (3) missing small weeds that germinated after we mapped the area.

The presence of weeds in agricultural fields can significantly impact crop yields, and farmers often rely on chemical herbicides to manage weed populations. However, weed resistance to herbicides is becoming increasingly common, and the effectiveness of chemical treatments can vary based on a variety of factors, such as weed species, growth stage, and environmental conditions^68^. Historical weed field conditions can also play a role, as previous management practices may have allowed for the buildup of weed seed banks in the soil. In the case of the SSWC and no-SSWC treatment plots, the higher weed presence in the SSWC plots could be attributed to a combination of factors, including spraying the herbicide too late for effective control, lack of overlap in herbicide application, and missing small weeds that germinated after the initial mapping. These findings highlight the importance of ongoing research and development of weed management strategies to address these challenges and improve crop yield and quality.

We do acknowledge that implementing the pipeline for SSWC presented on this paper requires specialized equipment, software, and knowledge in remote sensing and precision agriculture. It may not be feasible for all farmers to run this pipeline on their own, as it involves high initial investment in equipment such as drones and cameras, and requires technical expertise to operate the equipment and analyze the data. Additionally, the cost of acquiring and processing the high-resolution imagery may also be a limiting factor for some farmers. However, farmers could potentially hire service providers or companies that offer remote sensing and precision agriculture services to run the pipeline for them. This could be a more cost-effective and efficient solution for farmers looking for more sustainable solutions for weed control in their corn fields.

## Conclusion and future suggestions

In this study, we proposed a new approach of fusing unmanned aerial system (UAS) data with a spraying platform to minimize chemical use in weed control application for corn production. The results showed that our site-specific weed control (SSWC) approach has the potential to reduce financial expenditure in chemicals to apply a blanket application of herbicide in corn production. Moreover, our approach could produce healthier corn by using fewer chemicals. By identifying corn rows and removing them from the UAS imagery, we demonstrated an effective way to map weeds during the early growing season of corn. However, the human intervention required for weed mapping in this study would take approximately a day to collect and process data for making a weed prescription map, which may not be considered real-time prescription. Because of the significant reduction of chemical usage, our approach of spraying chemical herbicide only to those area where there are weeds could help to improve sustainability on corn production systems. Since 75% of all planted acres of cropland across the US are row crops, the approach we adopted in this study could be a foundational study for performing SSWC in the vast majority of US row crops.

To make our SSWC approach more user-friendly and consumer grade for commercial agricultural production, we suggest automating real-time prescription map generation from the information collected using UAS to reduce data collection and processing time. One potential solution for this is the use of embedded platforms to integrate UAS data with sprayer platform, which could be a field to explore in the near future. In addition, the overlap between individual nozzles of the sprayer should be set in a way that the spray-pattern covers the entire region set in the prescription map. Furthermore, the sprayer’s GPS refreshing rate needs improvement to operate at a higher speed for commercial farming practices. While the current study required a day to collect and process data to generate the prescription map, future research should aim to reduce the time required for real-time prescription mapping. By doing so, we could provide farmers and researchers with an efficient and effective way to manage weeds in corn production systems. Overall, the SSWC approach offers a promising alternative to conventional weed control methods in corn production, and we believe that our future suggestions could make it more accessible and useful for commercial agricultural production.

While investigating the potential effects of SSWC on grain yield is an important issue, it should be noted that this study was performed on “silage” corn, which is primarily used for livestock feed. As such, the impact of SSWC on grain yield was not be the primary focus of this study, but rather on reducing the use of chemical herbicides and improving the precision of weed control to achieve more sustainable and efficient agricultural practices. It is also important to consider the overall sustainability of agricultural production, which takes into account economic, environmental, and societal factors, rather than just focusing on grain yield alone.

## Data Availability

The UAS imagery and corn row detection technique (CRI algorithm) is available at: https://github.com/Ranjan1012/Crop-row-detection Shapefiles of orthophoto and experimental workflow is available at: https://drive.google.com/file/d/1dFgH79WTQ5Jh8AMzfNksciTMA9tIJ8XC/view?usp=sharing.

## References

[1] Feedgrains sector at a glance. https://www.ers.usda.gov/topics/crops/corn-and-other-feedgrains/feedgrains-sector-at-a-glance/ (2021).

[2] Theng D (2017). Fiberboards made from corn stalk thermomechanical pulp and kraft lignin as a green adhesive. BioResources.

[3] Garcia-Lara S, Serna-Saldivar SO, Serna-Saldivar SO (2019). Corn history and culture. Corn.

[4] Dhiman T, Satter L (1997). Yield response of dairy cows fed different proportions of alfalfa silage and corn silage. J. Dairy Sci..

[5] Pereira LG (2019). Comparison of biofuel life-cycle GHG emissions assessment tools: The case studies of ethanol produced from sugarcane, corn, and wheat. Renew. Sustain. Energy Rev..

[6] Jefferson M (2006). Sustainable energy development: Performance and prospects. Renew. Energy.

[7] Lal R (2015). Restoring soil quality to mitigate soil degradation. Sustainability.

[8] USGS. IHundreds of millions of pounds of pesticides are applied to agricultural crops every year to control weeds, insect infestations, plant diseases, and other pests (2017).

[9] 2021 pesticides in the pantry: Transparency & risk in food supply chains. https://www.asyousow.org/reports/2021-pesticides-pantry (2022).

[10] Sharma A (2019). Worldwide pesticide usage and its impacts on ecosystem. SN Appl. Sci..

[11] Örlander G, Nilsson U, Hällgren J (1996). Competition for water and nutrients between ground vegetation and planted *Picea abies*. NZJ For. Sci..

[12] Pakdaman Sardrood B, Mohammadi Goltapeh E, Lichtfouse E, Navarrete M, Debaeke P (2018). Weeds, herbicides and plant disease management. Sustainable Agriculture Reviews 31.

[13] Fugère V (2020). Community rescue in experimental phytoplankton communities facing severe herbicide pollution. Nat. Ecol. Evol..

[14] Vieira BC (2020). Herbicide drift exposure leads to reduced herbicide sensitivity in *Amaranthus* spp. Sci. Rep..

[15] Koutros S (2013). Risk of total and aggressive prostate cancer and pesticide use in the agricultural health study. Am. J. Epidemiol..

[16] Polańska K, Jurewicz J, Hanke W (2013). Review of current evidence on the impact of pesticides, polychlorinated biphenyls and selected metals on attention deficit/hyperactivity disorder in children. Int. J. Occup. Med. Environ. Health.

[17] Yan D, Zhang Y, Liu L, Yan H (2016). Pesticide exposure and risk of Alzheimer’s disease: A systematic review and meta-analysis. Sci. Rep..

[18] Kaur K, Kaur R (2018). Occupational pesticide exposure, impaired DNA repair, and diseases. Indian J. Occup. Environ. Med..

[19] Jakuboski, S. The dangers of pesticides|green science|learn science at scitable (2011).

[20] Srivastav AL, Singh AK, Kharwar RN (2020). Chemical fertilizers and pesticides: Role in groundwater contamination. Agrochemicals Detection, Treatment and Remediation.

[21] Zhang L, Yan C, Guo Q, Zhang J, Ruiz-Menjivar J (2018). The impact of agricultural chemical inputs on environment: Global evidence from informetrics analysis and visualization. Int. J. Low Carbon Technol..

[22] Kumar R, Kumar R, Prakash O (2019). Chapter-5 the impact of chemical fertilizers on our environment and ecosystem. Chief Ed.

[23] Hasan MK, Shahriar A, Jim KU (2019). Water pollution in Bangladesh and its impact on public health. Heliyon.

[24] Eyhorn F (2019). Sustainability in global agriculture driven by organic farming. Nat. Sustain..

[25] Steward BL, Gai J, Tang L (2019). The use of agricultural robots in weed management and control. Robot. Autom. Improv. Agric..

[26] Peteinatos GG, Weis M, Andújar D, Rueda Ayala V, Gerhards R (2014). Potential use of ground-based sensor technologies for weed detection. Pest Manag. Sci..

[27] See & spray$$^{{\rm TM}}$$ ultimate targeted, in-crop spraying. https://www.deere.com/en/sprayers/see-spray-ultimate (2022).

[28] López-Granados F (2011). Weed detection for site-specific weed management: Mapping and real-time approaches. Weed Res..

[29] Jin X, Che J, Chen Y (2021). Weed identification using deep learning and image processing in vegetable plantation. IEEE Access.

[30] Herwitz S (2004). Imaging from an unmanned aerial vehicle: Agricultural surveillance and decision support. Comput. Electron. Agric..

[31] Huang H (2018). A fully convolutional network for weed mapping of unmanned aerial vehicle (UAV) imagery. PLoS ONE.

[32] Mani PK, Mandal D, Pathan SK, Tariq A (2021). Remote sensing and geographic information system: A tool for precision farming. Geospatial Technologies for Crops and Soils.

[33] Thorp K, Tian L (2004). A review on remote sensing of weeds in agriculture. Precis. Agric..

[34] López-Granados F (2016). Early season weed mapping in sunflower using UAV technology: Variability of herbicide treatment maps against weed thresholds. Precis. Agric..

[35] Torres-Sánchez J, López-Granados F, De Castro AI, Peña-Barragán JM (2013). Configuration and specifications of an unmanned aerial vehicle (UAV) for early site specific weed management. PLoS ONE.

[36] Mink R (2018). Multi-temporal site-specific weed control of *Cirsium arvense* (L.) Scop. and *Rumex crispus* L. in maize and sugar beet using unmanned aerial vehicle based mapping. Agriculture.

[37] Peña JM, Torres-Sánchez J, de Castro AI, Kelly M, López-Granados F (2013). Weed mapping in early-season maize fields using object-based analysis of unmanned aerial vehicle (UAV) images. PLoS ONE.

[38] Gao J (2018). Fusion of pixel and object-based features for weed mapping using unmanned aerial vehicle imagery. Int. J. Appl. Earth Obs. Geoinf..

[39] Sapkota B, Singh V, Cope D, Valasek J, Bagavathiannan M (2020). Mapping and estimating weeds in cotton using unmanned aerial systems-borne imagery. AgriEngineering.

[40] Rasmussen J (2019). Pre-harvest weed mapping of *Cirsium arvense* in wheat and barley with off-the-shelf UAVs. Precis. Agric..

[41] Louargant M (2018). Unsupervised classification algorithm for early weed detection in row-crops by combining spatial and spectral information. Remote Sens..

[42] Zisi T (2018). Incorporating surface elevation information in UAV multispectral images for mapping weed patches. J. Imaging.

[43] Rasmussen J, Nielsen J (2020). A novel approach to estimating the competitive ability of *Cirsium arvense* in cereals using unmanned aerial vehicle imagery. Weed Res..

[44] Jurado-Expósito M, de Castro AI, Torres-Sánchez J, Jiménez-Brenes FM, López-Granados F (2019). *Papaver rhoeas* L. mapping with cokriging using UAV imagery. Precis. Agric..

[45] Koul S, Singh D, Kumar V, Kant R (2021). Machine learning and deep learning in agriculture. Smart Agriculture: Emerging Pedagogies of Deep Learning, Machine Learning and Internet of Things.

[46] Liakos KG, Busato P, Moshou D, Pearson S, Bochtis D (2018). Machine learning in agriculture: A review. Sensors.

[47] Hunter JE, Gannon TW, Richardson RJ, Yelverton FH, Leon RG (2020). Integration of remote-weed mapping and an autonomous spraying unmanned aerial vehicle for site-specific weed management. Pest Manag. Sci..

[48] Jensen, T. A., Smith, B. & Defeo, L. F. *An Automated Site-Specific Fallow Weed Management System Using Unmanned Aerial Vehicles*. GRDC Grains Research Update in Goondiwindi, Qld (2020).

[49] Pang Y (2020). Improved crop row detection with deep neural network for early-season maize stand count in UAV imagery. Comput. Electron. Agric..

[50] Varela S (2018). Early-season stand count determination in corn via integration of imagery from unmanned aerial systems (UAS) and supervised learning techniques. Remote Sens..

[51] Ronchetti G, Mayer A, Facchi A, Ortuani B, Sona G (2020). Crop row detection through UAV surveys to optimize on-farm irrigation management. Remote Sens..

[52] De Castro AI (2018). An automatic random forest-OBIA algorithm for early weed mapping between and within crop rows using UAV imagery. Remote Sens..

[53] Torres-Sánchez J, Mesas-Carrascosa FJ, Jiménez-Brenes FM, de Castro AI, López-Granados F (2021). Early detection of broad-leaved and grass weeds in wide row crops using artificial neural networks and UAV imagery. Agronomy.

[54] Vong CN, Conway LS, Zhou J, Kitchen NR, Sudduth KA (2021). Early corn stand count of different cropping systems using UAV-imagery and deep learning. Comput. Electron. Agric..

[55] Bah MD, Hafiane A, Canals R (2019). CRowNet: Deep network for crop row detection in UAV images. IEEE Access.

[56] Osco LP (2021). A CNN approach to simultaneously count plants and detect plantation-rows from UAV imagery. ISPRS J. Photogramm. Remote Sens..

[57] Basso M, Pignaton de Freitas E (2020). A UAV guidance system using crop row detection and line follower algorithms. J. Intell. Robot. Syst..

[58] Wang L, Xiang L, Tang L, Jiang H (2021). A convolutional neural network-based method for corn stand counting in the field. Sensors.

[59] 2020 state agriculture overview. https://www.nass.usda.gov/Quick_Stats/Ag_Overview/stateOverview.php?state (2020).

[60] Basics of corn production in North Dakota. https://www.ag.ndsu.edu/publications/crops/basics-of-corn-production-in-north-dakota (2019).

[61] Kudsk P, Lawrence KC (2002). Optimising herbicide performance. Weed Management Handbook.

[62] Pannacci E, Graziani F, Covarelli G (2007). Use of herbicide mixtures for pre and post-emergence weed control in sunflower (*Helianthus annuus*). Crop Prot..

[63] Farming & crop protection herbicides. https://agriculture.basf.us/crop-protection/products/herbicides/verdict.html (2022).

[64] Stow D (2019). Illumination geometry and flying height influence surface reflectance and NDVI derived from multispectral UAS imagery. Drones.

[65] Casa, R. *et al.* UAV-based hyperspectral imaging for weed discrimination in maize. In *Precision Agriculture’19* 24–35 (Wageningen Academic Publishers, 2019).

[66] Pignatti, S. *et al.* Maize crop and weeds species detection by using UAV VNIR hyperpectral data. In *IGARSS 2019–2019 IEEE International Geoscience and Remote Sensing Symposium* 7235–7238 (IEEE, 2019).

[67] David, L. C. G. & Ballado, A. H. Vegetation indices and textures in object-based weed detection from UAV imagery. In *2016 6th IEEE International Conference on Control System, Computing and Engineering (ICCSCE)* 273–278 (IEEE, 2016).

[68] Owen MD, Zelaya IA (2005). Herbicide-resistant crops and weed resistance to herbicides. Pest Manag. Sci. Former. Pestic. Sci..

